# Hoverflies provide pollination and biological pest control in greenhouse-grown horticultural crops

**DOI:** 10.3389/fpls.2023.1118388

**Published:** 2023-04-12

**Authors:** Hui Li, Kris A. G. Wyckhuys, Kongming Wu

**Affiliations:** ^1^ State Key Laboratory for Biology of Plant Diseases and Insect Pests, Institute of Plant Protection, Chinese Academy of Agricultural Sciences, Beijing, China; ^2^ Guangdong Laboratory for Lingnan Modern Agriculture, Guangzhou, China

**Keywords:** pollination, *Eupeodes corollae*, aphid control, horticulture, protected cultivation, agroecology, food safety

## Abstract

Beneficial insects provide pollination and biological control in natural and man-made settings. Those ecosystem services (ES) are especially important for high-value fruits and vegetables, including those grown under greenhouse conditions. The hoverfly *Eupeodes corollae* (Diptera: Syrphidae) delivers both ES, given that its larvae prey upon aphid pests and its adults pollinate crops. In this study, we investigated this dual role of *E. corollae* in three insect-pollinated and aphid-affected horticultural crops i.e., tomato, melon and strawberry within greenhouses in Hebei province (China). Augmentative releases of *E. corollae* increased fruit set and fruit weight of all three crops, and affected population dynamics of the cotton aphid *Aphis gossypii* (Hemiptera: Aphididae). On melon and strawberry, *E. corollae* suppressed *A. gossypii* populations by 54-99% and 50-70% respectively. In tomato, weekly releases of 240 *E. corollae* individuals/100 m^2^led to 95% fruit set. Meanwhile, releases of 160 hoverfly individuals per 100 m^2^led to 100% fruit set in melon. Also, at hoverfly/aphid release rates of 1:500 in spring and 1:150 in autumn, aphid populations were reduced by more than 95% on melon. Lastly, on strawberry, optimum levels of pollination and aphid biological control were attained at *E. corollae* release rates of 640 individuals/100 m^2^. Overall, our work shows how augmentative releases of laboratory-reared hoverflies *E. corollae* can enhance yields of multiple horticultural crops while securing effective, non-chemical control of resident aphid pests.

## Introduction

Pollination determines plant fitness, genetic diversity, and the overall functioning and long-term stability of the world’s ecosystems (Biesmeijer et al., 2006; Potts et al., 2010; Neuschulz et al., 2016). In agricultural production systems, insects or wind either transfer pollen grains naturally or human-assisted pollination can be implemented (Strange, 2015; Toni et al., 2021); these pollination processes are worth 9.5% of the total value of food production globally (Gallai et al., 2009; Breeze et al., 2011; Hanley et al., 2015). Insect-mediated pollination in particular ensures the sexual reproduction and primary productivity of multiple fruits, vegetables, oil crops, cereal grains and forages (Eilers et al., 2011; Jauker et al., 2012; Strange, 2015). More than 1,500 crops are pollinated by insects and these entomophilous plants produce 35% of the world’s food items (Klein et al., 2007; Aizen et al., 2009). Moreover, insect pollination routinely improves fruit quality and increases farmer incomes (Eilers et al., 2011; Chaplin-Kramer et al., 2014). Pollinating insects such as bees, flies, beetles, moths and butterflies thus generate economic dividends worth US$ 780.8 billion in 2016 globally (Rader et al., 2020). In different production systems e.g., almond or fruit orchards, or greenhouse vegetables, farmers fortify insect-mediated pollination through the establishment of rented or purchased hives of European honeybee (*Apis mellifera* L.) or bumblebees *Bombus* spp. (Roubik, 2002; Velthuis and Van Doorn, 2006; Potts et al., 2016).

In many farming systems, beneficial insects also provide a second ecosystem service i.e., biological pest control (Oerke, 2006; Power, 2010) which is valued at US$ 4.5 billion per year in the U.S. alone (Losey and Vaughan, 2006). In greenhouse settings, crop productivity is regularly affected by pestiferous herbivores such as aphids, thrips, whiteflies, and mites (Malais and Ravensberg, 2003; Maleknia et al., 2016). Aphids are common pests of greenhouse crops, where they reduce yields through direct (phloem) feeding, excretion of sugar-rich liquids that impede photosynthesis and the vectoring of debilitating viruses. Though chemical insecticides are routinely used for aphid control, these products cause resistance development, negatively affect resident pollinator populations, pollute the environment and pose important food safety hazards (Devine and Denholm, 2009; Garibaldi et al., 2018; Wyckhuys et al., 2020). Biological control is an environmentally friendly alternative to insecticide-based approaches, and has been continually used and refined for nearly 2000 years (Bale et al., 2008; Heimpel and Mills, 2017). In greenhouse production systems across the globe, biological control has replaced chemical pest control over the past decades (Pilkington et al., 2010).

In China and across the globe, beneficial insects are increasingly deployed for pest, disease and weed management in protected and open-field farming systems alike. However, the joint deployment of insect pollinators and biological control agents can either lead to positive (synergies) or negative (trade-offs) effects on crop yield (Lundin et al., 2013; Sutter and Albrecht, 2016; Garibaldi et al., 2018). For example, insect pollinators and pest control act synergistically on red clover (*Trifolium pratense* L.) to produce higher seed yields (Lundin et al., 2013). Similarly, managed pollinators such as bumblebees often vector insect-killing or antagonistic fungi and thus simultaneously deliver pollination and pest or disease control services (Shafir et al., 2006; Kapongo et al., 2008). On the other hand, high pest loads i.e., low levels of biological control enhanced pollinator benefits for seed yield in oilseed rape (*Brassica napus* L.) (Bartomeus et al., 2015).

Given their variable impact on crop productivity and the additional costs associated with a simultaneous use of pollinators and natural enemies, this practice has only received marginal attention in greenhouse agriculture. Science however can unlock opportunities to tap the synergistic interactions between pollinators and natural enemies, as demonstrated by the successful use of bumblebee pollinators for thrips biological control in Canadian tomato and sweet pepper crops (Kapongo et al., 2008).

Syrphid flies (Diptera: Syrphidae) commonly forage in agricultural crops, where they simultaneously provide pollination and biological control services (Ssymank et al., 2008; Omkar, 2016; Dunn et al., 2020). Hoverfly adults feed on pollen and nectar, making them the world’s second most important pollinators after bees (Rotheray and Gilbert, 2011; Rader et al., 2016; Klecka et al., 2018; Wotton et al., 2019; Rader et al., 2020). Hoverfly-mediated pollination has been valued at US$ 300 billion per year (Food and Agriculture Organisation of the United Nations, 2017). Members of the Syrphinae subfamily have carnivorous larvae that prey upon aphids, lepidopteran larvae and other soft-bodied herbivores (Nelson et al., 2012; Ramsden et al., 2017; Bellefeuille et al., 2021; Li et al., 2021; Li and Wu, 2022). The vagrant hoverfly *Eupeodes corollae* is a widely distributed hoverfly species that naturally occurs in Central Europe, North Asia and North Africa (Schweiger et al., 2007; Djellab et al., 2019). Adults of *E. corollae* consume pollen and floral nectar of cultivated crops and deposit eggs on aphid-infected plants (Jauker and Wolters, 2008; Pekas et al., 2020; Moerkens et al., 2021; Jiang et al., 2022). This hoverfly species is easily reared under laboratory conditions, creating lucrative opportunities for augmentation biological control (Van Lenteren et al., 2018). Untilrecently, few scientific studies have assessed how *E. corollae* augmentative releases affect fruit set, aphid pest control or yield in greenhouse crops. Pioneering work in Europe however has revealed 88% higher yields on sweet pepper crops that were subject to *E. corollae* releases (Pekas et al., 2020).

In this study, we investigated the relative contribution of *E. corollae* to pollination and aphid biological control in three entomophilous crops in China i.e., tomato *Solanum lycopersicum* L. (Solanaceae), melon *Cucumis melo* L. (Cucurbitaceae), and strawberry *Fragaria ananassa* Duch. (Rosaceae). In local greenhouses, these crops are regularly affected by the cotton aphid *Aphis gossypii* Glover (Rondon et al., 2005). For varying hoverfly release rates, we assessed fruit set, *A. gossypii* population dynamics, and the number of *E. corollae* eggs and larvae on melon and strawberry plants. Our work defines and validates crop-specific augmentative release schemes for *E. corollae*, opening new vistas for biodiversity-driven pollination and pest management in multiple greenhouse crops.

## Materials and methods

### Insect and plants

During June 2018, *E. corollae* adults were collected from alfalfa flowers by sweep netting at the Langfang Experimental Station of the Chinese Academy of Agricultural Sciences (CAAS) in Hebei Province, China (39.53°CN, 116.70°CE). Upon transfer to the laboratory, hoverflies were reared by placing five adult pairs in 0.5 x 0.3 x 0.4 m insect cages (120 mesh; Beijing Luhebang Technology Development Co., LTD). Each colony was fed with 10% honey-water solution and a 3:1 mixture (by weight) of commercial rape and maize pollen in two 9 cm diam petri-dishes and 40 laboratory-grown broad bean *Vicia faba* L. plantlets infested with *Megoura japonica* Matsumura aphids. Bean plantlets also served as an oviposition substrate, and were sporadically removed from rearing cages to collect egg. Broad bean seeds were obtained from Sichuan Kexi Seed Industry Co. LTD, China. Upon egg incubation, syrphid larvae were fed with *M. japonica* on bean plantlets until pupation. Newly emerged adults were then transferred to a new cage for breeding and oviposition. A cotton aphid *A. gossypii* colony was maintained on *Cucurbita pepo* L. plantlets (4-leaf stage) that were changed every 7 days. Aphid and syrphid populations were kept in climate-controlled rooms at 25 ± 1°CC, 30-70% RH, and 16h L:8h D. Seedlings of tomato (Yibaifen variety, 3-leaf stage), melon (Lvbaoshi, 3-leaf stage) and strawberry (Tianbao, 4-leaf stage) were purchased from the Shandong Shouguang Seedling company, China.

### Experimental set-up

All experiments were conducted in 25 x 5 m plastic greenhouses at the Gengfeng ecological garden in Hebei Province, China. Greenhouses were equipped with anti-insect vents (25 x 0.5 m; 120 mesh) that were adjusted to control temperature and humidity. A temperature and humidity data logger (ZW720, Xuzhou Fara Electronic Technology Co., LTD.) was further positioned within each greenhouse throughout the experiment. Two weeks before seedling transplanting, soil was tilled, and 150 kg of bio-organic fertilizer (Digyuan, Leshan Digyuan Biological Technology Co., LTD.) and 5 kg of potassium sulfate compound fertilizer (Alliance, Shandong Alliance Compound Fertilizer Co., LTD.) were evenly applied to the soil. Before the onset of the experiment, greenhouses were fumigated for two successive days with isoprocarb and chlorothalonil fungicide (Anyang Ruize Pesticide Co., LTD., China). During the course of the experiment, fungicide applocations were made based upon the actual disease occurrence while no insecticides were used.

*Tomato pollination*. Trials were conducted in two greenhouses from March 2020 to July 2020 (spring) and from August 2020 to March 2021 (autumn). In each greenhouse, 32 rows of tomato plantlets were established at 0.6 m inter-row and 0.3 m within-row spacing. Drip irrigation pipes were laid in each row. The cultivated area was divided into 4 plots, each consisting of 8 rows of tomato plants covered with a 5 x 5 x 3 m walk-in screen cage (80 mesh). Within each row, 4 tomato plants were randomly selected, marked and numbered. To ensure fruit quality, a maximum of 4 flowers were kept on each tomato branch. During spring or autumn, a respective 16 fruits (on 4 branches) or 12 fruits (on 3 branches) were retained per plant.

During spring and autumn, pollination trials were conducted in a flowering tomato crop. In each plot, different numbers of recently emerged (less than 24 h old) *E. corollae* adults were released on a weekly basis. Specifically, hoverfly adults were placed in 0.5 x 0.3 x 0.4 m cages, hand-carried to the greenhouses and cages were then opened within the greenhouse. More specifically, release rates included 20, 40, 60, 80, 120 individual adults in each plot. Over the course of the experiment, 3 and 4 releases were conducted during autumn and spring respectively. In other plots, tomato plants were sprayed with hormones to induce fruit formation or individual tomato flowers were bagged. For the hormone treatment, flower buds were inserted in 10 x 6 cm transparent bags (200 mesh) to prevent pollination by other insects. When tomato flowers were in full bloom, bags were removed to treat the anther and stigma with hormone (Fruit King, 0.5g/L, Hebei Shijiazhuang Hongwei Agricultural Science and Technology Development Co., LTD) using a 500 ml watering can. Only one hormone application was made per flower, after which each treated flower was re-inserted in the mesh bag until fruiting. In the bagging treatment, individual flower buds were inserted into 10 x 6 cm transparent mesh bags (200 mesh) prior to flowering, in order to prevent pollination by *E. corollae* or other resident insects. For each plot and (marked) tomato plant, we calculated fruit set and assessed fruit weight following each hoverfly release. During the experiment, environmental conditions ranged between 13.5-38.9°C and 32.2-94.2% during spring, and 8.2-36.4°C, 39.5-86.3% RH during autumn. Per season, seven plots were subject to different hoverfly release or hormone application treatments and one plot was used as a control i.e., bagging treatment.

*Melon pollination and aphid biological control*. Trials were conducted in two greenhouses from July 2020 to November 2020 (autumn), and from April 2021 to July 2021 (spring). In each greenhouse, 32 rows of muskmelon were planted at 0.6 m inter-row and 0.4 m within-row spacing. The cultivated area was divided into 4 plots, each consisting of 8 successive rows of melon plants covered by a 5 x 5 x 3 m walk-in cage made of insect-proof nets (80 mesh). In each plot, 24 melon plants were randomly selected and numbered, and 10 A*. gossypii* individuals were gently transferred from laboratory-grown *C. pepo* plantlets to the top leaves of each melon plant using a brush. Aphid-infested leaves were then inserted into 18 x 10 cm transparent bags (200 mesh). When the first melon flowers appeared (~7 days following aphid infestation), different numbers of newly emerged hoverfly adults (less than 24 h old) were released from 0.5 x 0.3 x 0.4 m insect cage into each plot, allowing them to freely escape from the cage. More specifically, we released 5, 10, 20, 40 or 80 pairs of *E. corollae* adults per plot. In two other plots, hormones were applied or flower bagging was done as described above. Next, all female flowers except for the two oldest (i.e., basal) ones were systematically removed. We equally recorded fruit set of each marked plant in each plot. In spring and autumn, six plots were subject to different hoverfly release or hormone application treatments and one plot was used as a control i.e., bagging treatment.

Upon termination of the pollination trial (i.e., ~13 days after aphid inoculation), mesh bags were removed from the plants and hoverfly releases were repeated at the above release rates. Hoverfly adults were released every 6 days, in all plots except for the hormone treatment-which now served as a control in the biological control experiment - and the flower-bagging treatment. As such, different numbers of hoverflies were released in each plot on days 7, 13, 19, 25, and 31 following aphid inoculation. The experiment was conducted at 19.3-38.5°C and 24.1-98.5% RH during spring, and 8.0-35.1°C and 36.4-79.2% RH during autumn. In each plot, we weighed melon fruits upon harvest and recorded the number of aphids, and *E. corollae* eggs and larvae on the days that hoverfly releases were made. Next, we calculated aphid population growth in the control treatment and the percentage degree of hoverfly-mediated biological control at time x through the following formula (Chaplin-Kramer et al., 2013):


$$\begin{array}{l} \text{Aphid population growth rate }\left(\text{time }x\right)\\ \;={\text{ aphid abundance t}}_{\text{x}}\text{/aphid population }\\ \;{\text{abundance t}}_{0}\text{in control treatment} \end{array}$$

$$\begin{array}{l} \text{Biological control rate }\left(\text{time }x\right)=[1-(({\text{aphid abundance t}}_{\text{x}}\\ \;{\text{/aphid abundance t}}_{0}\text{in treatment})\\ \;/\text{aphid population growth rate }\left({\text{time}}_{\text{x}}\right))]\;x100\% \end{array}$$

*Strawberry pollination and aphid biological control*. Trials were conducted in two greenhouses from November 2020 to March 2021. In each greenhouse, 24 rows of strawberry plantlets were established at 0.8 m inter-row and 0.15 m within-row spacing. The cultivated area in each greenhouse was divided into 4 plots, each consisting of 6 rows covered by a 5 x 5 x 3 m walk-in screen cage (80 mesh). To stimulate flower development, LED lights (400 nm-750 nm, 36W/10m^2^) were affixed 1.8 m above the plant canopy. Lights were only turned during cloudy days, from 8:00 to 20:00. On each plot, 30 strawberry plants were randomly selected and numbered, and 20 A*. gossypii* individuals were gently transferred to each plant (9-leaf stage). Once plants initiated flowering (~5 days following aphid infestation), hoverflies were released at the following rates: 10, 20, 40 or 80 pairs of newly emerged adults per plot, along with one control treatment in which no releases were done and flower buds were inserted in 10 x 6 cm transparent bags (200 mesh) to prevent pollination by other insects. Four consecutive releases were done in the experimental plots, at 10-day intervals. On hoverfly release days, we recorded the number of flowers and fruits on marked plants in each plot and calculated the respective fruit set rates. Strawberry fruits were weighed at harvest. Every five days, we further counted aphids, *E. corollae* eggs and larvae on marked plants. Based upon these data, we calculated the degree of hoverfly-mediated biological control (%) as above. The experiment was conducted at 4.3-26.1°C and 32.0-90.1% RH. The experiment consisted of 5 different treatments i.e., 4 different *E. corollae* release rates plus a control, and was only conducted once over time.

### Statistical analysis

Fruit set, fruit weight and aphid or hoverfly larval abundance data were analyzed by One-way analysis of variance (ANOVA) or Student’s t-test. Prior to analysis, all data were checked for normality and heteroscedasticity. Where necessary, data were transformed to meet normality assumptions. Statistical analysis was conducted by SPSS 21.0 (IBM, Armink, NY) and images were plotted with SigmaPlot 12.5 (Systat Software, Inc., Germany).

## Results

**Figure 1:**
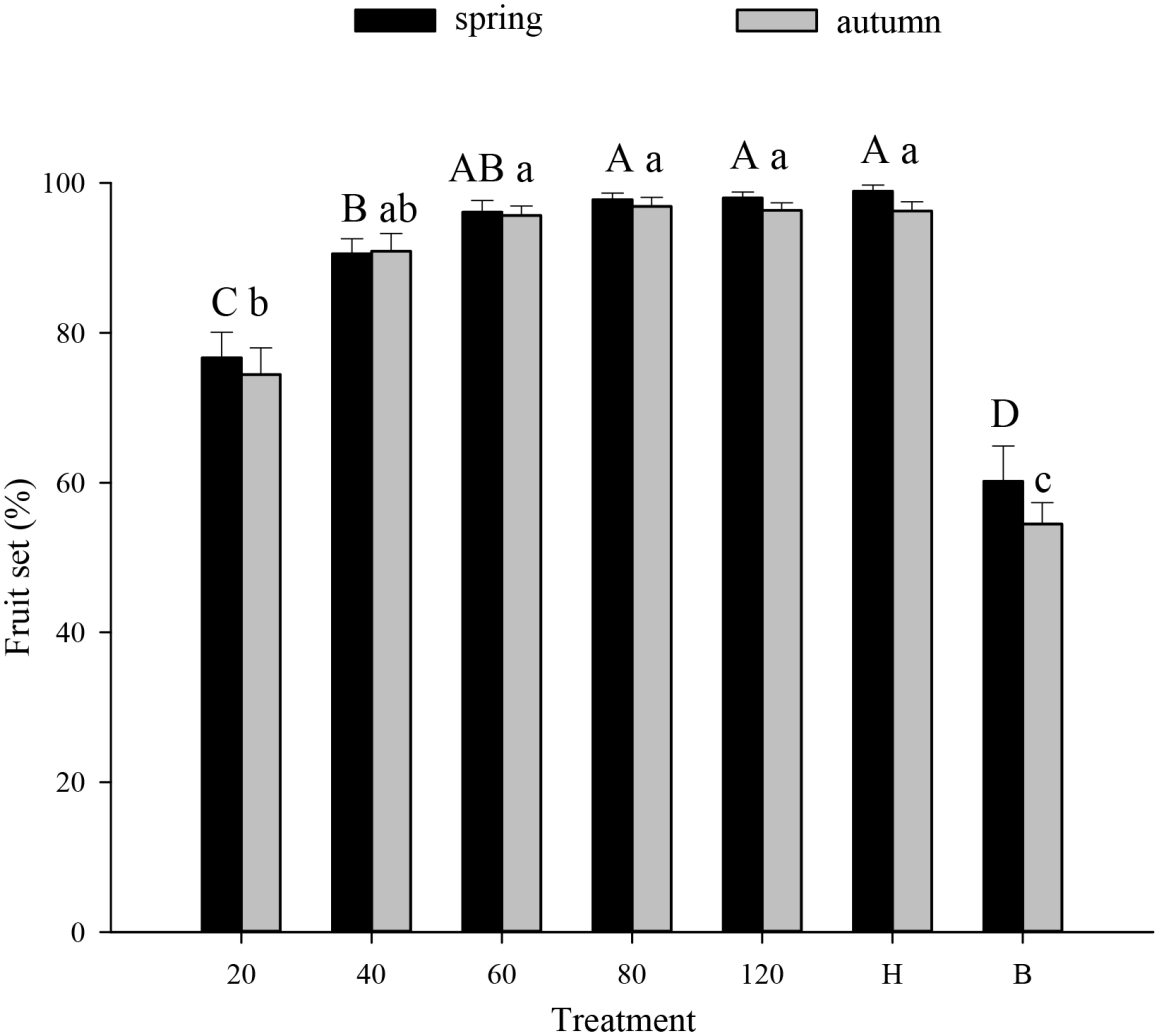
Fruit set of tomato (% per plant) under different experimental treatments during spring or autumn 2020-21. Numbers in the X axis refer to the total number of hoverfly adults that were released i.e., 20, 40, 60, 80 or 120; data are also plotted for hormone (H) and bagging (B) treatments. Different uppercase and lowercase letters indicate statistically significant differences between treatment groups during either season (One-way ANOVA, *P*<0.05, Tukey’s HSD *post-hoc*).

**Figure 2:**
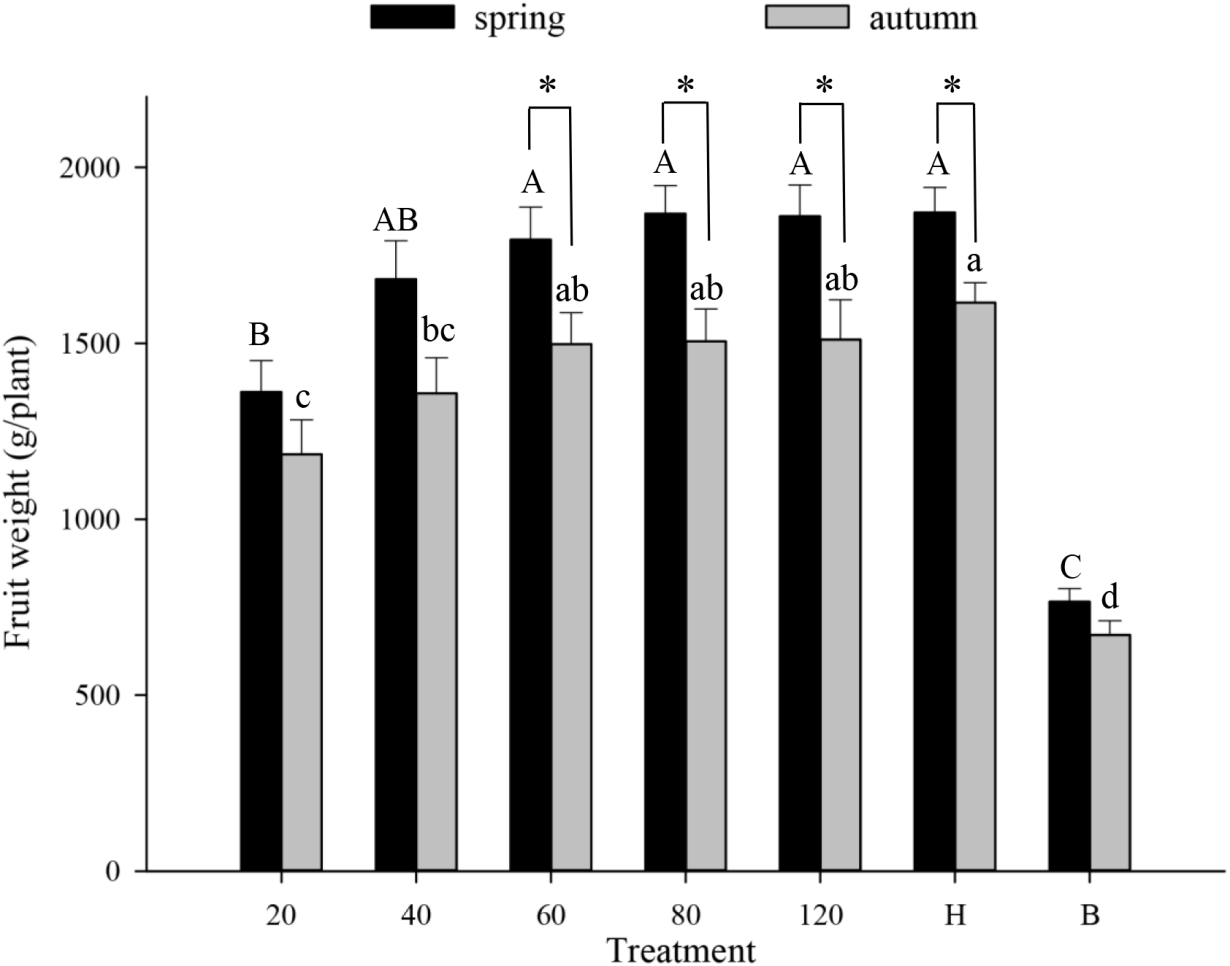
Fruit weight of tomato (g/plant) in different experimental treatments during spring and autumn 2020-21. Numbers in the X axis refer to the total number of hoverfly adults that were released i.e., 20, 40, 60, 80 or 120; data are also plotted for hormone (H) and bagging (B) treatments. Different uppercase and lowercase letters indicate statistically significant differences between treatment groups during either season (One-way ANOVA, *P*<0.05, Tukey’s HSD *post-hoc*). Asterisks refer to seasonal differences in fruit weight under the same treatment.

*Tomato pollination* Tomato fruit set was higher in plots that received hoverfly releases than in bagged plots. With increasing *E. corollae* release rate, fruit set initially increased but then remained constant during both spring and autumn (**Figure 1**). During spring, the highest fruit set (97.8%) was recorded in plots with releases of 80 *E. corollae* adults. Fruit set under this treatment was comparable to hormone-treated plants (98.9%), and markedly higher than for the bagging treatment (60.2%) (*F*_6,210 _= 36.73, *P*=0.000). During autumn, fruit set in plots with releases of 60 hoverfly adults (95.7%) did not differ from that of hormone-treated plants (96.3%) (*F*_1,60 _= 0.44, *P*=0.73). Similarly, the weight of harvested tomatoes was highest in plots that were subject to hoverfly releases. With increasing release rates, tomato weight initially increased and then remained unchanged during either season (**Figure 2**). During spring, a release rate of 60 hoverflies yielded a fruit weight of 1794.0 ± 92.4 g, which was identical to that of hormone-treated plants (1871.3 ± 71.1 g), but higher than for the bagging treatment (765.4 ± 37.2 g) (*F*_6,207 _= 34.1, *P*=0.000). For different hoverfly release rates i.e., 60, 80, 120 adults and the hormone treatment, fruit weight during spring was higher than during autumn (60: *F*_1,53 _= 0.52, *P*=0.032; 80: *F*_1,53 _= 0.1, *P*=0.005; 120: *F*_1,48 _= 0.14, *P*=0.018; and hormone: *F*_1,55 _= 1.1, *P*=0.008 respectively).

**Figure 3:**
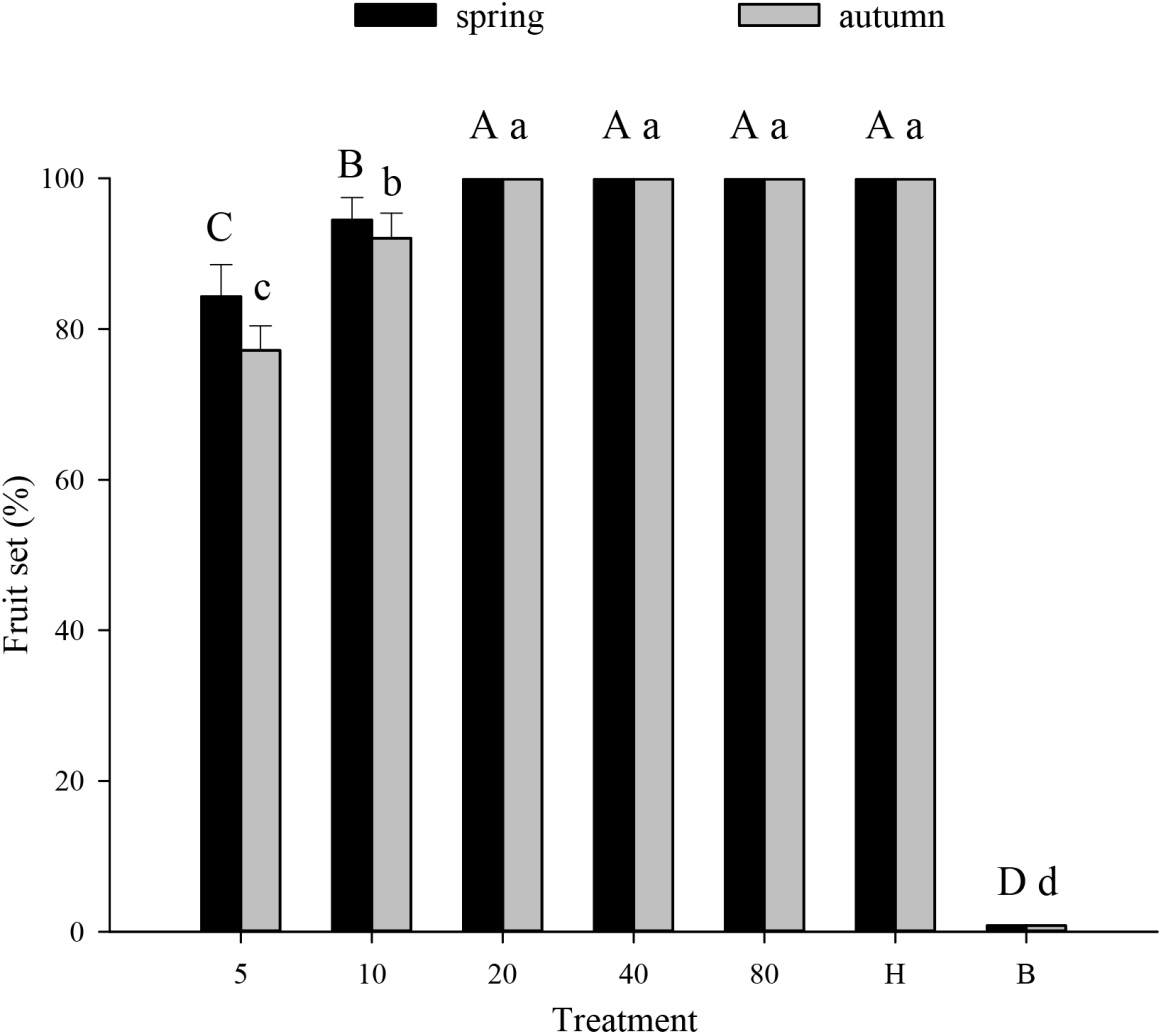
Fruit set of muskmelon (% per plant) under different experimental treatments during spring or autumn 2020-21. Numbers in the X axis refer to the total number of hoverfly adults that were released i.e., 5, 10, 20, 40 or 80 pairs; data are also plotted for hormone (H) and bagging (B) treatments. Different uppercase and lowercase letters indicate statistically significant differences between treatment groups during either season (One-way ANOVA, *P*<0.05, Tukey’s HSD *post-hoc*).

**Figure 4:**
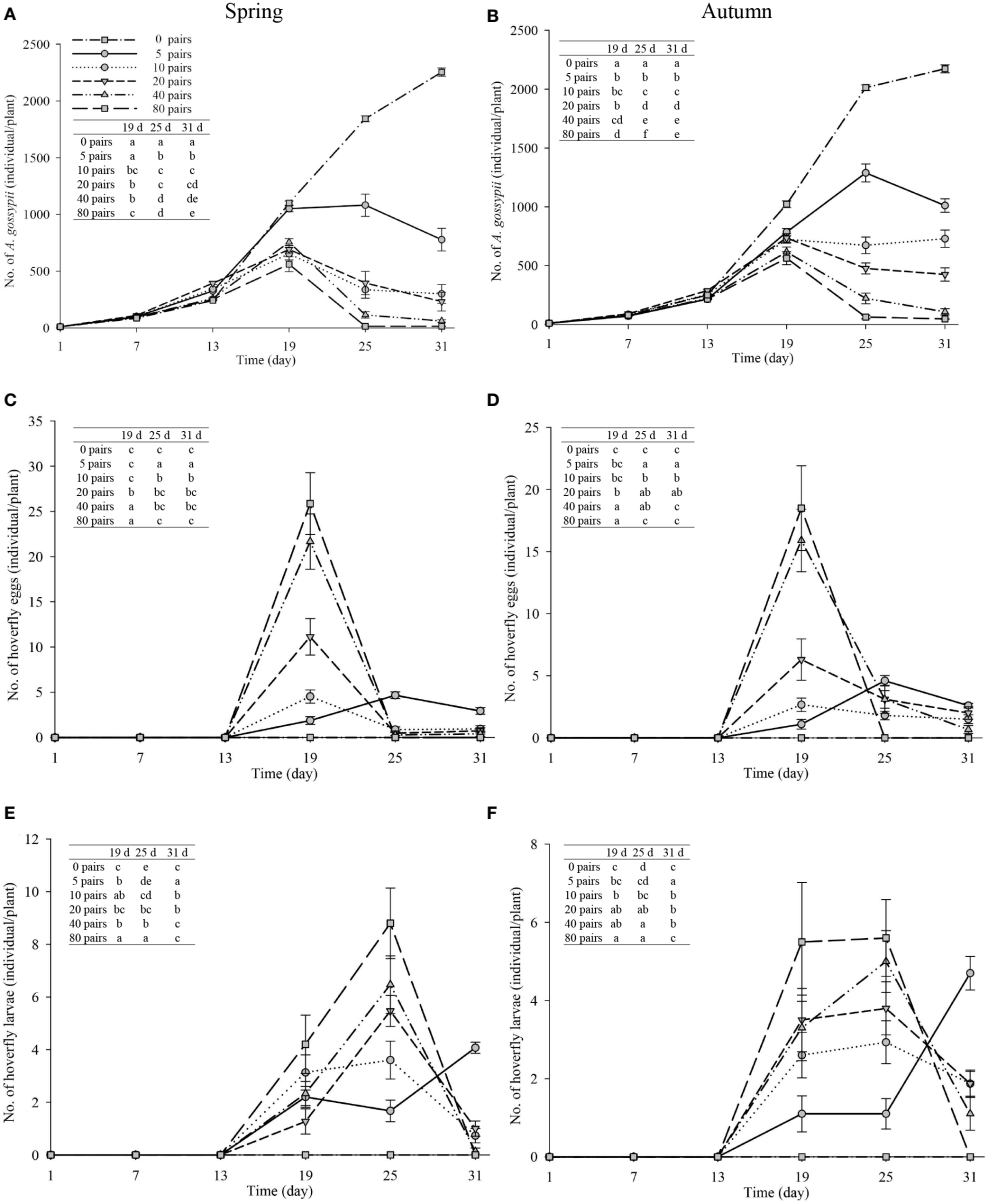
Number of *A. gossypii* **(A, B)**, hoverfly eggs **(C, D)** and hoverfly larvae **(E, F)** per muskmelon plant over time during spring and autumn 2020-21. Hoverflies were released on day 7, 13, 19, 25 and 31. Mesh bags that covered individual aphid-infested plants were removed on day #13. Data are plotted for different hoverfly release rates i.e., 0 (i.e., hormone treatment), 5, 10, 20, 40 and 80 adult pairs. Different lowercase letters indicate statistically significant differences between treatment groups on the same time in each figure (One-way ANOVA, *P*<0.05, Tukey’s HSD *post-hoc*).

*Melon pollination and biological control*. Melon fruit set was higher in plots that received hoverfly releases than in bagged plots. With increasing *E. corollae* release rate, fruit set initially increased and then remained unchanged during spring and autumn (**Figure 3**). Upon release of 20 *E. corollae* pairs, 100% fruit set was attained during either season; a rate identical to that of hormone-treated plants and substantially higher than for the bagging treatment (spring: *F*_6,157 _= 675.8, *P*=0.000; autumn: *F*_6,157 _= 415.6, *P*=0.000). For a given release rate, fruit set did not differ between spring and autumn season (**Figure 3**). During spring, aphid infestation levels declined at higher hoverfly release rates with the extent of *A. gossypii* population decline ranging from 65.5-99.3% (**Figure 4A**). Once mesh bags were removed (i.e., day #13), aphid population growth rate declined in the hoverfly release treatments and peak population numbers were reached on day #19. By the end of the experiment (i.e., day #31), aphid infestation levels were reduced by 65.5 ± 12.3%, 86.7 ± 8.8%, 89.7 ± 7.8%, 97.3 ± 4.2% and 99.3 ± 2.2% under the different hoverfly release treatments i.e., 5, 10, 20, 40, 80 pairs as compared to the 0 pairs (Hormone group) (**Figure 4A**). Similar results were obtained during autumn, with the extent of aphid population reduction directly proportional to hoverfly release rate (**Figure 4B**). However, the degree of population reduction was smaller than during spring, especially at low hoverfly release rates. On day #31, aphid population levels were reduced by 53.5 ± 12.9%, 66.5 ± 12.2%, 80.4 ± 10.2%, 95.0 ± 5.6% and 97.8 ± 3.8% as compared to the hormone group (**Figure 4B**). During either season, the number of *E. corollae* eggs on melon plants increased with the hoverfly release rate (**Figures 4C, D**). The number of hoverfly eggs on a single melon plant was highest on day #19. Under a given release treatment, egg deposition rates were higher during spring than autumn (5 pairs: *F*_1,46 _= 2.349, *P*=0.034; 10 pairs: *F*_1,46 _= 0.026, *P*=0.000; 20 pairs: *F*_1,39 _= 4.205, *P*=0.001; 40 pairs: *F*_1,45 _= 0.393, *P*=0.002; and 80 pairs: *F*_1,40 _= 1.452, *P*= 0.007).

**Figure 5:**
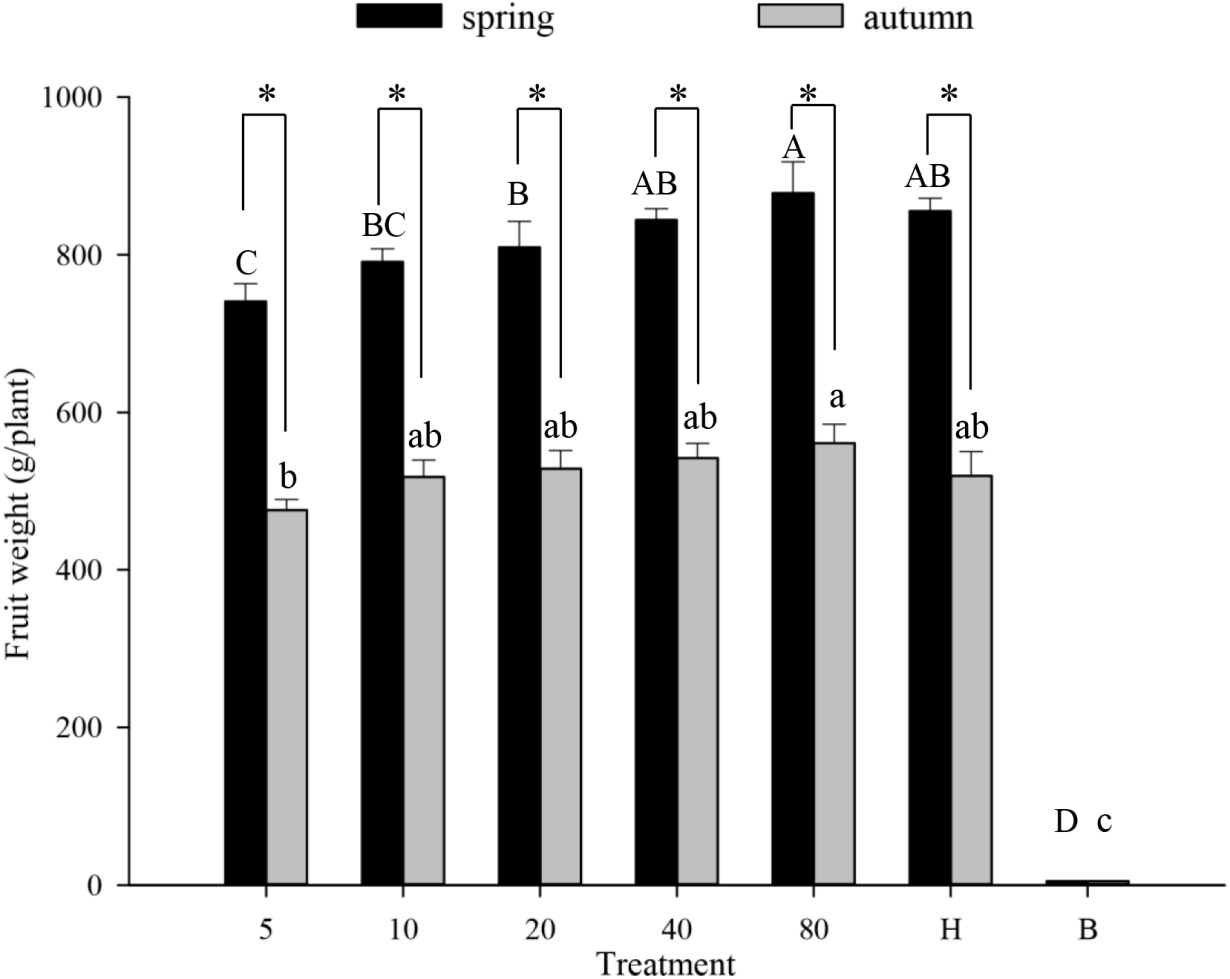
Fruit weight of muskmelon (g/plant) in different experimental treatments during spring and autumn 2020-21. Numbers in the X axis refer to the total number of hoverfly adults that were released i.e., 5, 10, 20, 40 or 80 pairs; data are also plotted for hormone (H) and bagging (B) treatments. Different uppercase and lowercase letters indicate statistically significant differences between treatment groups during either season (One-way ANOVA, *P*<0.05, Tukey’s HSD *post-hoc*). Asterisks refer to seasonal differences in fruit weight under the same treatment.

Similarly, the number of hoverfly larvae increased with the hoverfly release rate during either season (**Figures 4E, F**). Larval numbers were highest on day #25 in all treatments except for the lowest hoverfly release rate i.e., 5 pairs (**Figures 4E, F**). Under a given release treatment, hoverfly larvae were more abundant during spring than autumn (20 pairs: *F*_1,39 _= 0.001, *P*=0.005; 40 pairs: *F*_1,45 _= 0.424, *P*=0.007; and 80 pairs: *F*_1,40 _= 3.146, *P*=0.000 respectively). Lastly, during both seasons, melon weight increased proportionally with hoverfly release rate (**Figure 5**). Under a given release treatment, fruit weight was higher during spring than in autumn (**Figure 5**) and the highest melon weights were recorded for a release rate of 80 *E. corollae* pairs. The latter values i.e., 878.2 ± 39.7 g and 560.9 ± 24.2 g during spring or autumn, respectively were comparable to those of the hormone treatment i.e., 832.1 ± 11.6 g and 519.6 ± 30.7 g (**Figure 5**).

**Figure 6:**
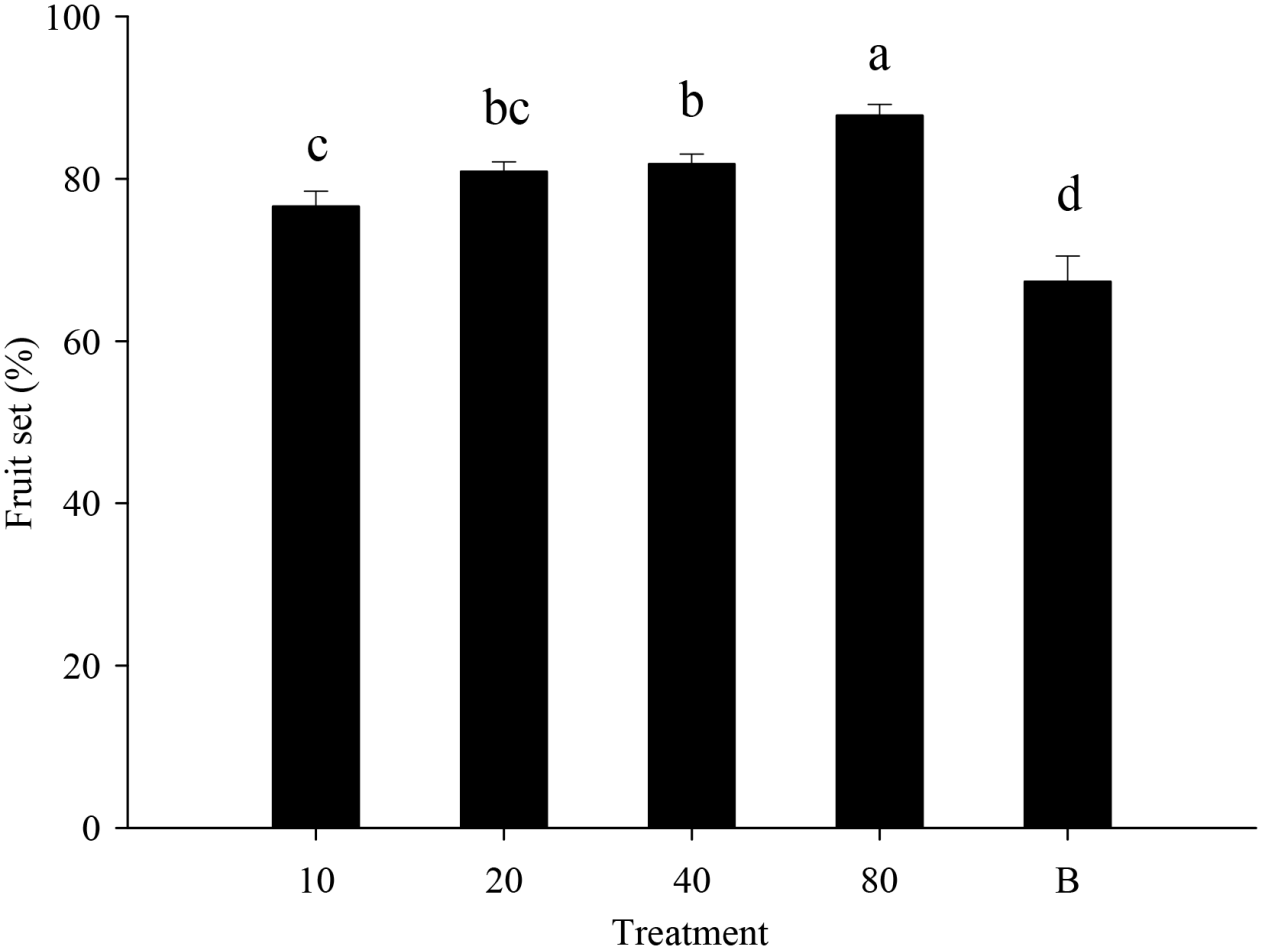
Fruit set of strawberry (% per plant) under different experimental treatments during winter 2020-21. Numbers in the X axis refer to the total number of hoverfly adults that were released i.e., 10, 20, 40 or 80 pairs; data are also plotted for a bagging (B) treatment. Different lowercase letters indicate statistically significant differences between treatment groups (One-way ANOVA, *P*<0.05, Tukey’s HSD *post-hoc*).

**Figure 7:**
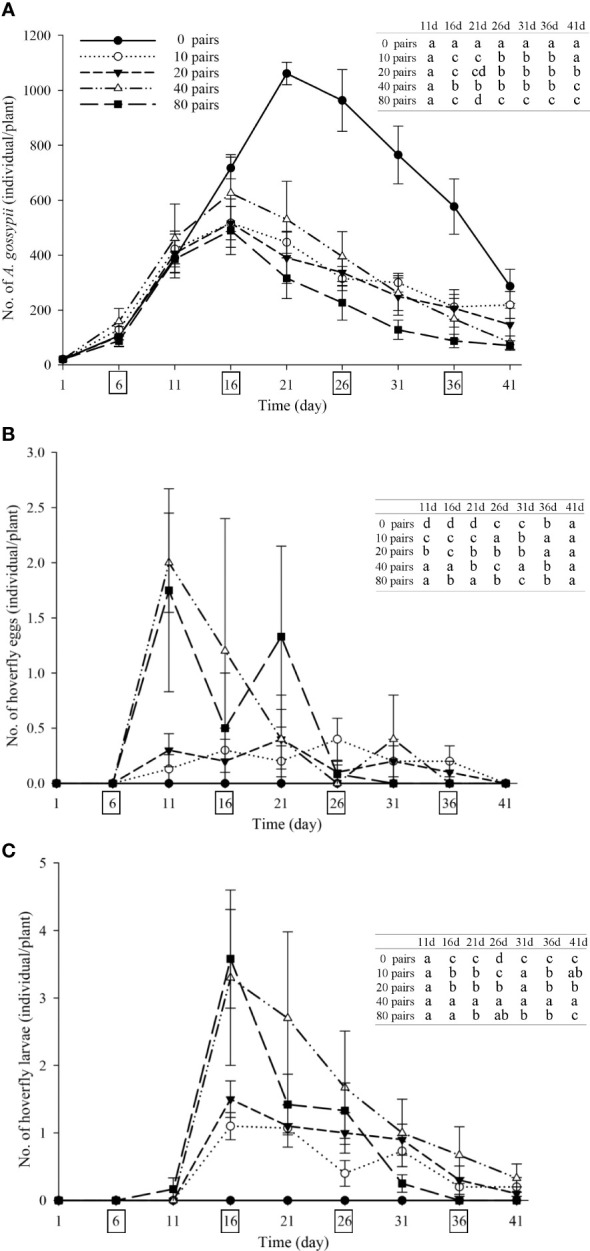
Number of *A. gossypii* **(A)**, hoverfly eggs **(B)** and hoverfly larvae **(C)** per strawberry plant over time during winter 2020-21. Four consecutive hoverfly releases were conducted (as shown by boxes on the abscissa in each figure). Data are plotted for different hoverfly release rates i.e., 0 (i.e., bagging treatment), 10, 20, 40 and 80 adult pairs. Different lowercase letters indicate statistically significant differences between treatment groups on the same time in each figure (One-way ANOVA, *P*<0.05, Tukey’s HSD *post-hoc*).

**Figure 8:**
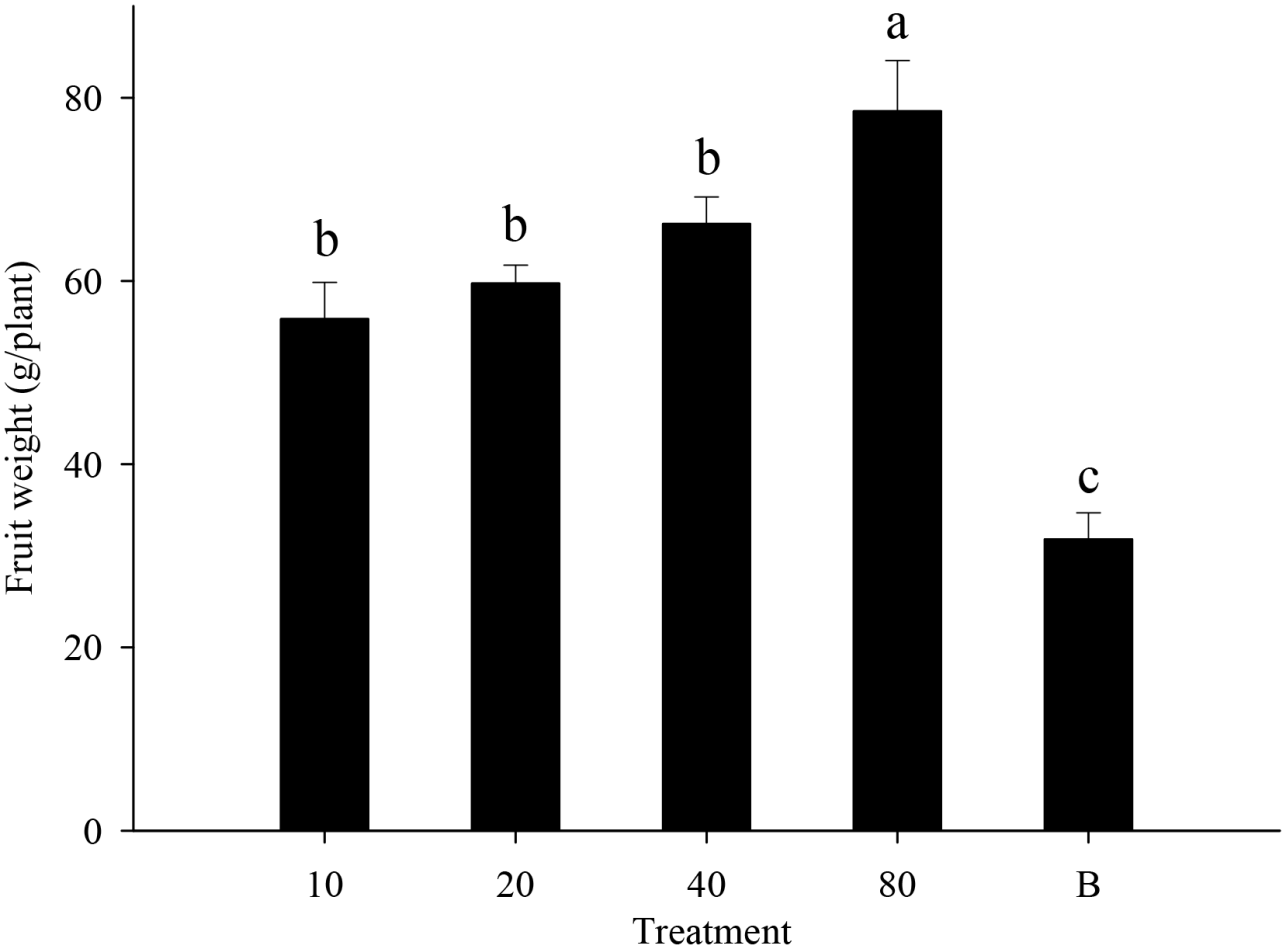
Fruit weight of strawberry (g/plant) in different experimental treatments during winter 2020-21. Numbers in the X axis refer to the total number of hoverfly adults that were released i.e., 10, 20, 40 or 80 pairs; data are also plotted for a bagging (B) treatment. Different lowercase letters indicate statistically significant differences between treatment groups (One-way ANOVA, *P*<0.05, Tukey’s HSD *post-hoc*).

*Strawberry pollination and biological control*. Strawberry fruit set was higher in plots that received hoverfly releases than in bagging plots, and increased with release rate. Fruit set under release treatments ranged from 76.6-87.8%, and was markedly higher than for bagged plants i.e., 67.3% (*F*_4,148 _= 13.7, *P*=0.000) (**Figure 6**). Aphid populations were suppressed under all hoverfly release schemes and the lowest *A. gossypii* abundance was recorded for release rates of 80 pairs (**Figure 7A**). As compared to the bagged treatment where *A. gossypii* attained peak abundance of 1061.3 ± 40.9 individuals per plant on day # 21, aphid population growth slowed markedly following hoverfly release. Under release rates of 10, 20, 40 or 80 *E. corollae* pairs, aphid population levels were reduced by a respective 57.9 ± 12.7%, 63.2 ± 12.5%, 50.0 ± 12.9% and 70.2 ± 11.8% as compared to the bagging group. Under release rates of 40 and 80 *E. corollae* pairs, high numbers of eggs were deposited on strawberry plants during two weeks following hoverfly release (**Figure 7B**). Similarly, the number of hoverfly larvae was highest on day #16 following release, attaining respective maxima of 1.07 ± 0.2, 1.5 ± 0.27, 3.3 ± 1.3 and 3.58 ± 0.73 larvae per plant (**Figure 7C**). Lastly, the highest strawberry weight (78.5 ± 5.5 g/plant) was recorded for a release rate of 80 pairs; this weight surpassed that for other release rates or for the bagging treatment i.e., 31.8 ± 2.9 g (**Figure 8**; *F*_4,148 _= 14.485, *P*=0.000).

## Discussion

Syrphid flies, such as *E. corollae*, provide globally important ecosystem services such as pollination and biological control in both natural and agricultural ecosystems. Aside from being a widely-distributed pollinator, *E. corollae* larvae consume aphid pests and lepidopteran larvae in various agricultural crops and agro-ecosystems (Hopper et al., 2011; Li et al., 2021). Pioneering work in Europe has shown how laboratory-reared *E. corollae* effectively deliver non-chemical pest control and enhance fruit set in sweet pepper (Pekas et al., 2020). Building upon these initial findings, we demonstrate how *E. corollae* augmentative releases slow aphid pest build-up and raise yield levels in other greenhouse crops i.e., tomato, muskmelon and strawberry. Even at small release rates, considerable increments in fruit set, fruit weight and aphid biological control rates were observed for all three crops. Considering how multiple hoverfly species can be reared under laboratory conditions (Iwai et al., 2007; Iwai et al., 2009), our work unlocks opportunities for augmentative hoverfly releases in (greenhouse) cropping systems in China and abroad.

Though we investigated hoverfly-mediated pollination and aphid pest control on muskmelon and strawberry, we were unable to assess the latter service on tomato crops. Upon inoculation of tomato plants with different aphid species (*A. gossypii*, *Myzus persicae* (Sulzer), *Aphis craccivora* Koch), these failed to colonize the plants. This can possibly be ascribed to the effect of glandular trichomes on tomato leaves (Blanco-Sánchez et al., 2021), which inhibit aphid colonization and feeding. Tomatoes are a self-pollinating plants that produce many flowers and exhibit a long flowering period. Wind and insect vectors promote pollen deposition, fertilization and fruit set through at a low rate. In greenhouses, hormones and honeybees or bumblebees are often used to promote tomato fruit set (Sabara and Winston, 2003; Higo et al., 2004). Pollination is thus a crucial factor in commercial tomato production, and hoverfly releases improve crop output. Also, as *E. corollae* larvae do not consume common tomato pests such as thrips and whiteflies (Bleeker et al., 2009; Gupta et al., 2018), hoverfly adults did not forage or oviposit on (flowerless) tomato plants in our trials. Hence, in the sole presence of tomato, *E. corollae* populations likely cannot sustain themselves in greenhouse systems and may require recurrent adult releases.

As compared to the European or North American horticultural sector, Chinese producers commonly use low-tech plastic (vs. glass) greenhouses with limited or no climate control. As our study mimicked these conditions, temperatures occasionally reached peaks of 38-39°CC i.e., as recorded in the upper half of the closed structure. Such elevated temperatures may have negatively affected behavior, development and overall fitness of the study organisms i.e., aphids and hoverflies. Those unfavorable climatic conditions possibly explain some of the differences in hoverfly or aphid population dynamics or fruit yield between spring and autumn seasons i.e., in muskmelon and tomato. Notably, hoverfly egg and larval densities on muskmelon were substantially lower during autumn. This however may also be attributed to shortened daylength or cooler night-time temperature. Overall, it is challenging to fully ascertain the magnitude of those effects as laboratory-derived temperature sensitivity measures do not fully account for hoverfly behavioral adaptations under field conditions (e.g., Hassall et al., 2017). Yet, lower *E. corollae* reproductive output during autumn is plausibly mirrored in dampened aphid suppression at intermediate release rates, and such needs to be taken into account in future biological control endeavors.

In order to optimally gauge *E. corollae* effects on strawberry pollination, we opted not to employ fruit thinning. As a result, we attained up to 88% fruit set and 10-15 fruits per plant at release rates of 80 hoverfly pairs. This practice however contrasts with commercial strawberry cultivation in which thinning is routinely used to improve fruit quality. Given that *E. corollae* releases concurrently enhance fruit set and fruit weight, this practice potentially results in higher overall yields at lower thinning rates and could be of major interest to producers. Our novel experimental set-up further allowed to distinguish hoverfly impacts on pollination vs. aphid pest control, and thus provides advantages over a simple determination of yield (Hodgkiss et al., 2018). Aside from providing direct aphid control, pollinators such as *E. corollae* also selectively favor direct or indirect plant defence in a strawberry crop (Egan et al., 2021) and thereby enhance further opportunities for sustainable pest management (Wyckhuys et al., 2022).

Aphids often co-occur with other greenhouse pests such as whiteflies, thrips, mites. As *E. corollae* does not necessarily prey upon all of these other pests, hoverfly releases can easily be combined with other (augmentative, conservation) biological control measures or preventative pest management. Living and UV-reflective mulches can be deployed against thrips and whitefly pests, while natural enemies such as *Phytoseiulus persimilis* Athias-Henriot and *Diglyphus isaea* (Walker) have been effectively used against mites and agromyzid leafminers (Nyoike and Liburd, 2010; Park and Lee, 2021). Aphids and thrips can be simultaneously controlled with coordinated releases of *Aphidius colemani* Viereck and *Neoseiulus cucumeris* Oudemans, while a combination of hoverfly releases with bee-vectored microbials carries ample potential for multi-target pest or pathogen control (Kapongo et al., 2008; Messelink et al., 2013; Gonzalez et al., 2016). Yet, when combining hoverflies with other (invertebrate, microbial) natural enemies under integrated control programs, hoverfly release rates and multi-species augmentation schemes will need to be defined based upon ecological interactions and overall cost-effectiveness. Especially when tailored extension services are lacking, smallholders may consider it impractical to use multiple natural enemies and might still resort to pesticide applications. Several of the insecticides that are commonly used against whitefly or thrips however cause important lethal and sublethal effects on hoverflies (Colignon et al., 2003; Moens et al., 2011). Hence, an effective integration of hoverfly-mediated services with chemical pest control is only possible for a narrow set of selective chemical compounds (Jansen et al., 2011; Moens et al., 2011). A judicious use of chemical insecticides and their proper timing, dosage and placement is thus of paramount importance to integrated pest management (IPM) success.

Aside from its poor compatibility with chemical control, economic aspects may also hamper a further diffusion of hoverfly augmentative releases especially in low-value crops. In order to incentivize and accelerate its adoption, comprehensive benefit-cost analyses, fine-tuned release schemes and multi-stakeholder communication is crucial. This can comprise a full accounting of the (human, environmental) health benefits of foregone insecticide applications (Wyckhuys et al., 2020). Habitat management tactics can also improve economic viability. Costly, repetitive releases of laboratory-reared hoverflies possibly can be avoided through the use of banker plants, as demonstrated for the hoverfly *Eupeodus americanus* (Wiedemann) in Canadian sweet pepper (Miller and Rebek, 2018; Bellefeuille et al., 2021). For example, legume companion plants can provide alternative aphid prey and sustain viable hoverfly populations within greenhouse settings (Tscharntke et al., 2005; Winqvist et al., 2011). In order to enhance hoverfly fitness and stimulate oviposition, plants that provide alternative, non-pest prey can be paired with ones that bear copious amount of pollen or (floral, extra-floral) nectar (Campbell et al., 2012; Laubertie et al., 2012; Martínez-Uña et al., 2013; Gurr et al., 2017). To pinpoint the most appropriate species of nectar-producing plants, banker plants and non-target aphids that can accompany *E. corollae* augmentative releases, further-study is imperative.

In conclusion, our work uncovers how *E. corollae* hoverflies assume a dual role as pollinators and biological control agents in three different greenhouse crops. Our research shows that augmentative hoverfly releases augment fruit set and crop yield while securing insecticide-free aphid pest control. To fully exploit the benefits of hoverflies for (greenhouse) horticulture in China and abroad, follow-up research is required to define (economically sound) release schemes, assess habitat management tactics and explore a further integration with other (insect-vectored) natural enemies.

## Data availability statement

The original contributions presented in this study are included in the article/supplementary material. Further inquiries can be directed to the corresponding author.

## Author contributions

HL: Investigation, Formal analysis, Writing-original draft. KAW: Writing - review and editing. KW: Funding acquisition, Conceptualization, Supervision, Resources, Writing - review and editing. All authors contributed to the article and approved the submitted version.
